# SUMOylation of DRIL1 Directs Its Transcriptional Activity Towards Leukocyte Lineage-Specific Genes

**DOI:** 10.1371/journal.pone.0005542

**Published:** 2009-05-14

**Authors:** Alexandre Prieur, Karim Nacerddine, Maarten van Lohuizen, Daniel S. Peeper

**Affiliations:** Department of Molecular Genetics, The Netherlands Cancer Institute, Amsterdam, The Netherlands; Ordway Research Institute, United States of America

## Abstract

DRIL1 is an ARID family transcription factor that can immortalize primary mouse fibroblasts, bypass RAS^V12^-induced cellular senescence and collaborate with RAS^V12^ or MYC in mediating oncogenic transformation. It also activates immunoglobulin heavy chain transcription and engages in heterodimer formation with E2F to stimulate E2F-dependent transcription. Little, however, is known about the regulation of DRIL1 activity. Recently, DRIL1 was found to interact with the SUMO-conjugating enzyme Ubc9, but the functional relevance of this association has not been assessed. Here, we show that DRIL1 is sumoylated both *in vitro* and *in vivo* at lysine 398. Moreover, we provide evidence that PIASy functions as a specific SUMO E3-ligase for DRIL1 and promotes its sumoylation both *in vitro* and *in vivo*. Furthermore, consistent with the subnuclear localization of PIASy in the Matrix-Associated Region (MAR), SUMO-modified DRIL1 species are found exclusively in the MAR fraction. This post-translational modification interferes neither with the subcellular localization nor the DNA-binding activity of the protein. In contrast, DRIL1 sumoylation impairs its interaction with E2F1 *in vitro* and modifies its transcriptional activity *in vivo*, driving transcription of subset of genes regulating leukocyte fate. Taken together, these results identify sumoylation as a novel post-translational modification of DRIL1 that represents an important mechanism for targeting and modulating DRIL1 transcriptional activity.

## Introduction

E2FBP1/ARID3A/DRIL1 (hereafter referred to as DRIL1) was originally isolated as a novel E2F1 heterodimeric partner that stimulates E2F-dependent transcription [1]. DRIL1 belongs to the AT-rich interaction domain (ARID) family DNA-binding protein known to exert pleiotropic roles in embryonic patterning, cell lineage gene regulation, cell cycle control, chromatin remodeling and transcriptional regulation [2]. DRIL1 is evolutionary conserved, with specific orthologs in the fly, mouse, zebrafish and C. elegans genomes [2]. Drosophila DRIL1 (DRI) is developmentally regulated, being expressed in a restricted population of cells including a subset of neural cells of the central nervous system, differentiating cells of the gut, and salivary gland ducts [3]. DRI is involved in various developmental processes, either as a transcriptional activator or repressor. Its loss-of-function mutations result in embryonic lethality [4]. Experiments in Xenopus embryos identified DRIL1 as a novel regulator of TGFβ signaling and a vital component of mesodermal patterning and embryonic morphogenesis [5]. The mouse ortholog of DRIL1, B cell regulator of Ig heavy chain transcription (BRIGHT), exhibits specific matrix-attachment regions (MARs) binding properties and regulates immunoglobulin transcription at late stages of B lymphocyte differentiation [6]. BRIGHT protein was found to associate with Bruton's tyrosine kinase (Btk) [7]. Defective Btk causes inherited agammaglobulinemia, an X-linked severe immunodeficiency characterized by an early block in B cell differentiation at the pre-B to immature B cell stage, resulting in abnormal low levels of serum Ig [8]. While murine BRIGHT protein seems to be prevalently found in the B cell-lineage, human DRIL1 expression appears to be ubiquitous, raising the possibility that the function of DRIL1 depends on the species and the cellular context [9]. We recently demonstrated that DRIL1 allows primary mouse fibroblasts (MEFs) to efficiently bypass both spontaneous and activated RAS-induced senescence by deregulating the Rb/E2F1 pathway [10]. On the other hand, DRIL1 was shown to be a direct p53 target gene and to be activated in response to specific cues, such as UV-induced DNA damage or doxorubicin treatment in a p53-dependent manner [11]. The functional connection of DRIL1 to both Rb/E2F1 and p53 tumor suppressor pathways, as well as its pivotal function in promoting either cellular proliferation or growth arrest, implies a tight and finely tuned regulation of DRIL1 activity and binding affinities. However, such regulatory mechanisms of DRIL1 function remain largely unknown.

Post-translational modification of transcription factors is a common and important mechanism to achieve dynamic regulation of gene expression. Small Ubiquitin-related MOdifier (SUMO) has emerged as an important post-translational regulator involved in a wide range of cellular processes including gene transcription, cell cycle progression, genomic integrity and chromatin dynamics [12]. Sumoylation is a multistep enzymatic reaction in which SUMO is activated by an E1 Aos1/Uba2 heterodimer in an ATP-dependent manner, transferred to E2-conjugating enzyme Ubc9 and covalently linked to target lysine residues of protein substrates *via* an isopeptide bond. Ubc9 interacts directly with the substrates and is able to append SUMO to lysine residues *in vitro*. However, different classes of SUMO E3 ligases, including the nucleoporin RanBP2, the protein inhibitor of activated STAT (PIAS) family, and Polycomb Pc2, have been shown to considerably enhance the sumoylation efficiency by bridging SUMO-Ubc9 to specific target proteins [12]. Conjugation to SUMO is highly dynamic and reversible; hence, several desumoylating enzymes (also named Ubiquitin-like proteases) are involved in the removal of SUMO moiety [13].

Interestingly, DRIL1 has been shown previously to interact directly with Ubc9 and Sp100, a scaffold component of the PML nuclear bodies (NBs, also referred to as PODs, ND10s) [14]. Nuclear bodies are dynamic macromolecular domains known to favor the docking of SUMO-modified proteins [15]. These results, together with the recent findings showing that RBP1, an ARID-containing protein, undergoes SUMO-dependent regulation of its repressor activity [16], prompted us to investigate whether DRIL1 is subjected to SUMO modification and whether sumoylation contributes to the regulation of DRIL1 transcriptional activity.

We observed that DRIL1 is covalently modified by SUMO1 *in vitro* and *in vivo* at a single lysine residue identified as lysine 398. The nuclear matrix-associated SUMO E3 ligase PIASy, but not other PIAS proteins nor RanBP2, serves as a SUMO E3 ligase for DRIL1 by efficiently enhancing its sumoylation *in vitro* and *in vivo*. Consistent with the subnuclear localization of PIASy, SUMO-modified DRIL1 forms are found exclusively in the MAR fraction. A SUMO-deficient DRIL1 mutant is not subject to incorrect subcellular localization and retains DNA-binding capability comparable to wild-type DRIL1. In contrast, DRIL1 sumoylation prevents it from interacting with E2F1 and appears to redirect its transcriptional activity towards leukocyte lineage-specific genes. Altogether, this study establishes sumoylation of DRIL1 as a novel and relevant control mechanism modulating DRIL1 function.

## Results

### DRIL1 is sumoylated *in vitro* and *in vivo*

**Figure 1:**
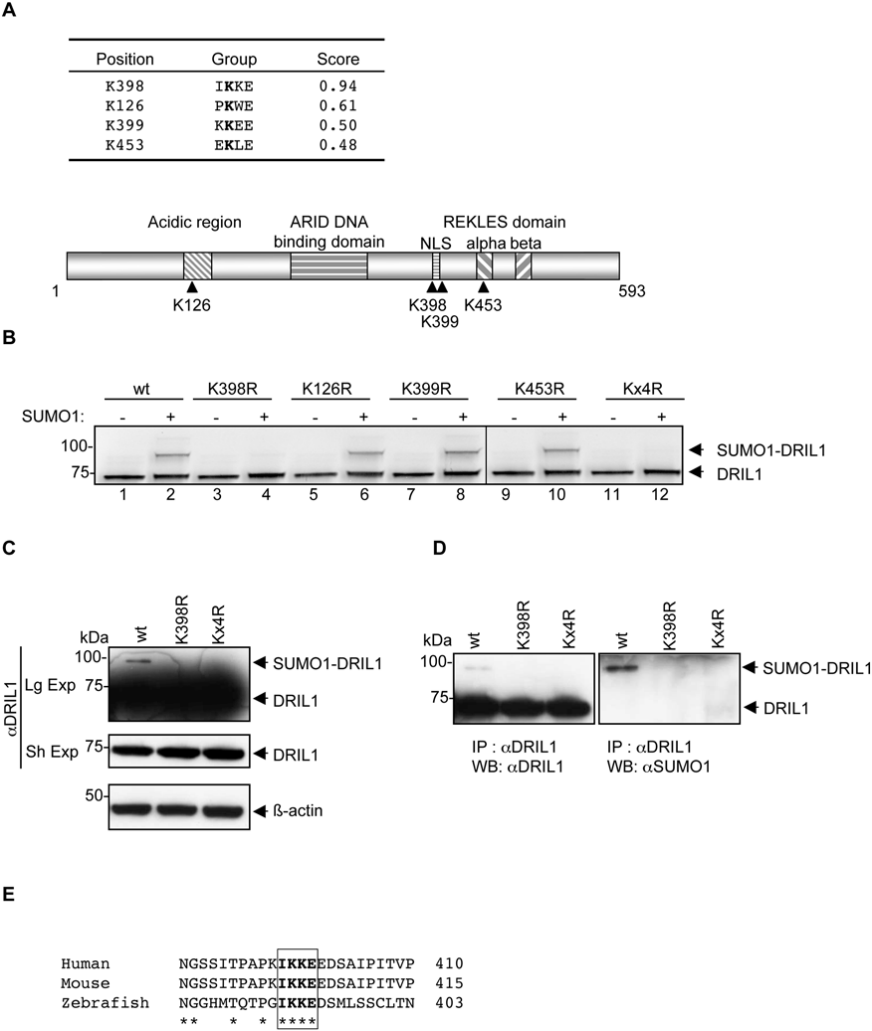
DRIL1 is sumoylated *in vitro* and *in vivo*. (A) Table showing DRIL1 potential SUMO consensus motifs and SUMOplot™ score (Abgent; www.abgent.com/doc/sumoplot). Below is the schematic structure of DRIL1 showing lysines 126, 398, 399, 453 and the functional domains. (B) *In vitro* sumoylation assay performed with ^35^S-labeled *in vitro*-translated wt, SUMO point mutants (K126R, K398R, K399R, K453R) or the quadruple mutant (Kx4R) of DRIL1, incubated in a sumoylation mix containing purified E1, E2 and ATP in the absence or presence of SUMO1. (C) 293T cells transfected with wt DRIL1, K398R or Kx4R mutants. Lysates were Western blotted using antibodies against DRIL1 and β-actin as loading control. (Sh Exp) for short exposure and (Lg Exp) for long exposure. (D) 293T cells transfected with wt DRIL1, K398R or Kx4R. Lysate were immunoprecipited (IP) with antibody against DRIL1, and precipited proteins were western blotted (WB) using antibodies against DRIL1 (left panel) or SUMO1 (right panel). (E) Alignment of amino acid sequences of DRIL1 from human, mice and zebrafish spanning the conserved SUMO consensus motif using Clustal software.

The sumoylated target lysine is usually found in the so-called SUMO consensus motif consisting in a ΨKXE/D tetrapeptide, where ‘Ψ’ denotes an hydrophobic residue, and ‘X’ any residue [12]. In order to examine if DRIL1 is a potential target for sumoylation, we analyzed its amino acid sequence with the SUMOplot™ Analysis Program (Abgent; www.abgent.com/doc/sumoplot). The sequence scan of DRIL1 protein identified four potential SUMO consensus motifs with significant SUMOplot™ scores (Fig. 1A, upper part), raising the possibility that DRIL1 can be sumoylated. The four putative target lysine residues, K126, K398, K399 and K453, appear to be scattered throughout the DRIL1 protein (Fig. 1A, lower part). In addition to the central ARID core, DRIL1 consists of an acidic N-terminal region of unknown function that includes K126, and an oligomerization domain, named REKLES (after this conserved hexa-aminoacid motif), in the C-terminal third of the molecule in which K453 resides [2]. Lysines 398 and 399 are located in a putative nuclear localization signal (NLS).

To address experimentally whether DRIL1 is modified by SUMO, we used an *in vitro*-reconstituted sumoylation assay, in which a ^35^S-labeled DRIL1, generated by *in vitro* translation, serves as a target for SUMO modification in the presence of recombinant E1 (the Aos1/Uba2 heterodimer), recombinant Ubc9, SUMO1, and ATP. Following the reaction, the proteins were resolved by SDS-PAGE and visualized by autoradiography. In the control reaction, which lacked SUMO1, a unique and expected band of DRIL1 migrating at 75 kDa was detected (Fig. 1B, lane 1), whereas an additional +20 kDa shifted band was observed when recombinant SUMO1 was added to the *in vitro* reaction, at the predicted size of a SUMO1-DRIL1 conjugate (Fig. 1B, lane 2), indicating that DRIL1 is sumoylated *in vitro*. Then, we assayed lysine to arginine substitution mutants (K126R, K398R, K399R, K453R and the quadruple mutant Kx4R) for their ability to be sumoylated *in vitro*. When Kx4R and K398R mutants were tested, or any double and triple mutant that includes the K398R substitution, sumoylation was completely abolished (Fig. 1B, lanes 4, 12 and data not shown). These results indicated that DRIL1 sumoylation occurs on K398 and this lysine is the unique SUMO acceptor site in the *in vitro* conditions.

Next, we addressed whether DRIL1 is also able to undergo SUMO modification in a cellular context. Therefore, we transfected plasmids expressing wild type (wt) DRIL1, K398R and Kx4R mutants into 293T cells. Western blotting of cell extracts revealed a 75 kDa molecular weight protein corresponding to DRIL1, as well as a slower migrating SUMO1-DRIL1 conjugated species migrating at 95 kDa (Fig. 1C). Importantly, this band was not observed when either K398R or Kx4R SUMO mutant was expressed, suggesting that, in intact cells also, K398 is the exclusive target lysine for modification by the endogenous SUMO machinery.

To address if this slower migrating band corresponds to a SUMO1-DRIL1 species, we immunoprecipitated DRIL1 from these lysates using DRIL1 antibody, and analyzed the precipitates by western blotting using DRIL1 and SUMO1 antibodies. As shown in figure 1D, the slower migrating form of DRIL1 was recognized by the anti-SUMO1 antibody, confirming that DRIL1 is conjugated to SUMO1 *in vivo*. Importantly, K398R and Kx4R mutations completely suppressed DRIL1 sumoylation since neither the anti-DRIL1 nor the anti-SUMO1 antibody detected a 95 kDa product (Fig. 1D). Collectively, these experiments show that DRIL1 is sumoylated *in vivo* and that lysine 398 is the unique site for this modification. Alignment of the amino acid sequences between human, mouse and zebrafish DRIL1 orthologs revealed that K398 and the residues that form the SUMO consensus motif are highly conserved, suggesting that sumoylation of DRIL1 is a mechanism maintained throughout evolution (Fig. 1E).

### PIASy is a SUMO E3 ligase for DRIL1

**Figure 2:**
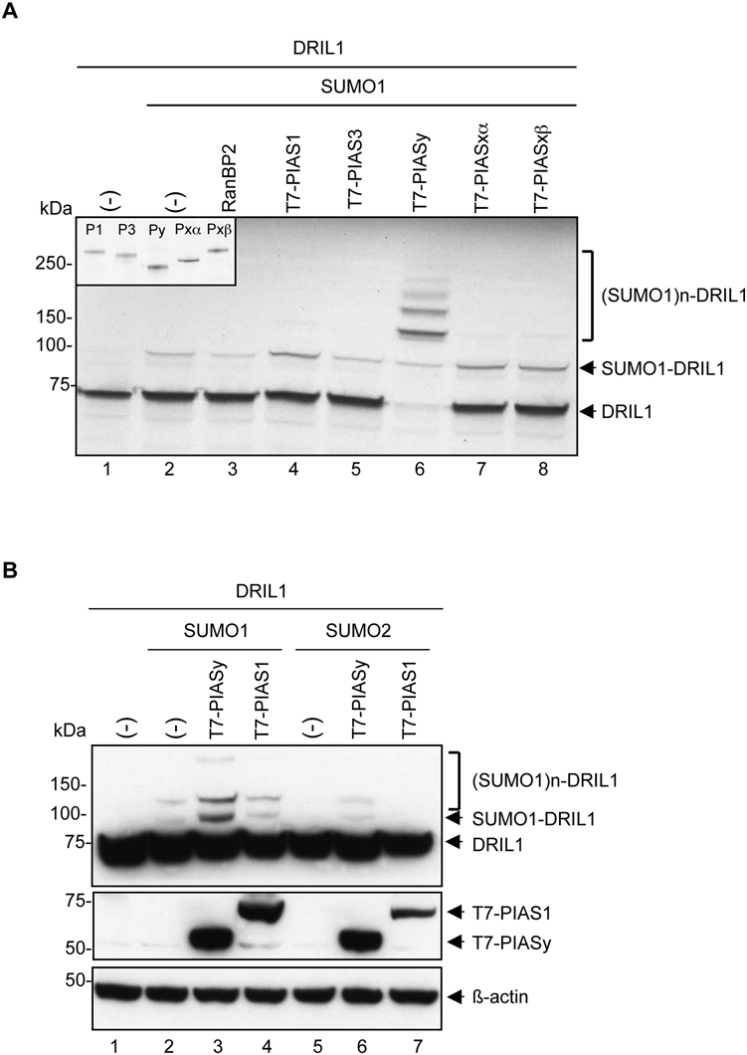
PIASy is an E3 SUMO ligase for DRIL1. (A) *In vitro* sumoylation assay performed with ^35^S-labeled *in vitro*-translated wt DRIL1, *in vitro*-translated PIAS1, PIAS3, PIASy, PIASxα, PIASxβ or recombinant RanBP2 and incubated in a sumoylation mix containing purified E1, E2, ATP and SUMO1. 10% of the *in vitro*-translated PIAS1 (P1), PIAS3 (P3), PIASy (Py), PIASxα (Pxα), PIASxβ (Pxβ) were ^35^S-labeled *in vitro*-translated for input control (inset). (-) for empty vector. (B) 293T cells co-transfected with wt DRIL1, T7-PIAS1, T7-PIASy, SUMO1 or SUMO2 constructs. Lysates were Western blotted using antibodies against DRIL1, T7 and β-actin as loading control. (-) for empty vector.

In reconstituted sumoylation assays, E3 ligases are dispensable, but in a physiological context these proteins play an essential role in regulating post-translational sumoylation of proteins. Few proteins have been identified to function as SUMO E3 ligases. They include the members of the PIAS protein family, the nucleoporin RanBP2 and the Polycomb group protein Pc2 (also CBX4). Since DRIL1 K398 is located in a putative NLS and DRIL1 is a MAR binding protein, we hypothesized that either the nucleoporin RanBP2 or the MAR-associated PIASy protein may function as E3 ligase for DRIL1. Therefore, we tested RanBP2 and PIAS family members for their potency to catalyze DRIL1 sumoylation *in vitro*. When SUMO1 was added to the reaction mix, only minimal sumoylation of DRIL1 was observed after 15 minutes of assaying (Fig. 2A, lane 2). Addition of recombinant RanBP2 active fragment (lacking the FG repeats, also termed RanBP2ΔFG) or unlabeled *in vitro* translated PIAS3 to the SUMO reaction had no effect on the reaction (Fig. 2A, lanes 3, 5), while addition of PIAS1, PIASxα and PIASxβ induce only a moderate increase in DRIL1 sumoylation (Fig. 2A, lanes 4,7,8). In contrast, addition of PIASy to the SUMO reaction dramatically enhanced DRIL1 sumoylation, leading to the complete disappearance of the unmodified form of DRIL1 and its conversion into poly-sumoylated products (Fig. 2A, lane 6). This was not due to any differential expression of the PIAS proteins (Fig. 2A, insert). These results show that PIASy displays efficient and specific SUMO ligase activity towards DRIL1.

Next, we investigated whether PIASy protein exerts similar activity towards DRIL1 *in vivo*. 293T cells were co-transfected with DRIL1, PIASy and SUMO1 expression constructs. As shown in figure 2B, PIASy expression significantly enhanced SUMO modification of DRIL1 (lane 3). Similarly to what was observed *in vitro*, PIASy induced the attachment of at least three SUMO1 molecules to DRIL1. This could either represent SUMO–SUMO chains that are formed on lysine 398, the major SUMO attachment site in DRIL1, or may result from SUMO conjugation to additional – less potent acceptor - lysine residues of DRIL1. In contrast, and also consistent with the results obtained *in vitro*, PIAS1 overexpression had no effect on DRIL1 sumoylation and cells exhibited comparable (basal) levels of SUMO1-DRIL1 to that observed in control SUMO1 co-transfected cells (Fig. 2B compare lane 2 with lane 4). Interestingly, PIASy-mediated DRIL1 sumoylation was specific for SUMO1, as it was not observed for the SUMO2 paralogue (Fig. 2B, lanes 5, 6 and 7). Hence, although we cannot formally exclude the possibility that other PIAS proteins can promote DRIL1 sumoylation in certain tissue or cellular contexts, these data demonstrate that PIASy is a bona fide, potent and specific SUMO E3 ligase for DRIL1 *in vitro* and *in vivo*, and exerts a selective activity in promoting DRIL1 modification by SUMO1.

### Sumoylation does not affect DRIL1 subcellular localization

**Figure 3:**
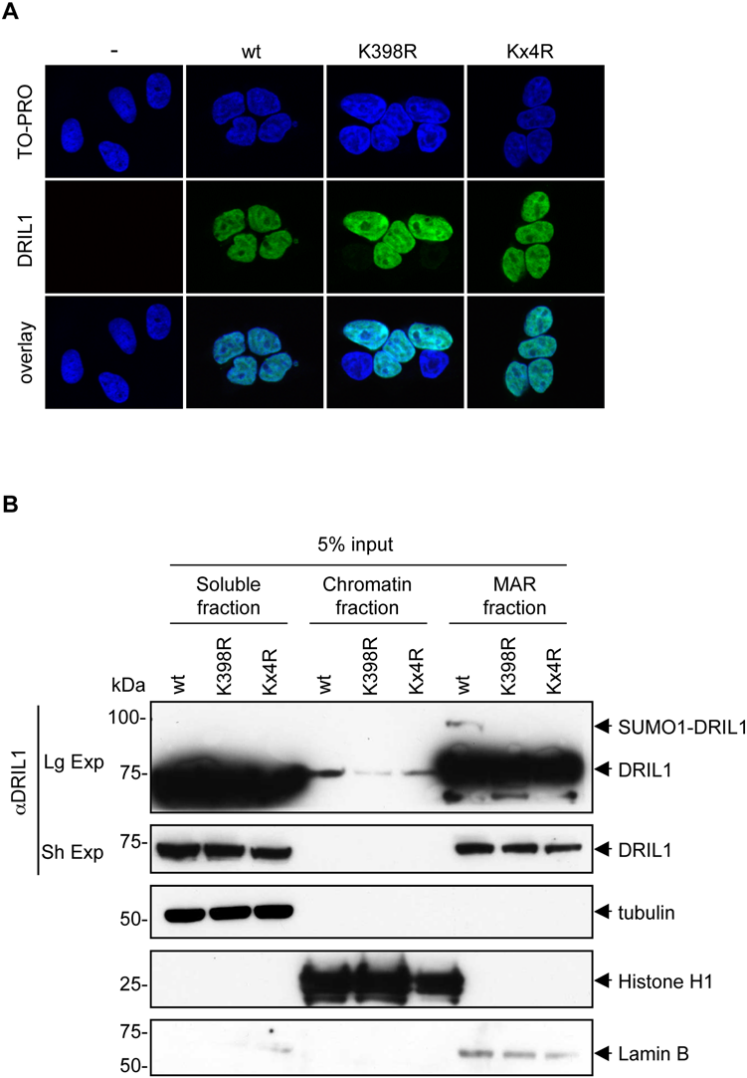
Sumoylation does not affect DRIL1 localization. (A) Immunofluorescence of DRIL1 in 293T cells transfected with wt DRIL1, K398R or Kx4R. DNA was counterstained with TO-PRO. (B) wt DRIL1, K398R or Kx4R were expressed in 293T cells and fractions were prepared as described in “materials and methods”. Relative nuclear distribution of DRIL1 was determined by SDS-PAGE and western blotting. Purity of fractions was assessed by blotting with antibodies against tubulin (soluble), histone H1 (chromatin) and lamin B (matrix associated region, MAR). (Sh Exp) for short exposure and (Lg Exp) for long exposure.

In addition to other functions, sumoylation has been linked to multiple aspects of nucleocytoplasmic trafficking and subnuclear targeting of substrates [12]. Previous work has shown that the murine ortholog of DRIL1, named BRIGHT, resides mainly within the nuclear matrix but is able to actively shuttle between the nucleus and the cytoplasm in a Crm1- and cell cycle-dependent manner. This is mediated by a functional nuclear localization signal (NLS) and nuclear export signal (NES) within the REKLES region [17]. To address whether SUMO1 modification is required for proper subcellular localization of DRIL1, we compared the localization of wt DRIL1 with the sumoylation-deficient mutants. 293T cells were transfected with wt DRIL1 or K398R, and analyzed by indirect immunofluoresence and confocal microscopy. As shown in figure 3A, wt DRIL1 and SUMO-defective mutant showed a similar pattern of intranuclear localization, suggesting that SUMO modification is not essential for DRIL1 cellular localization to the nucleus. Importantly, the Kx4R mutant was also found correctly localized to the nucleus, ruling out a role for any of these candidate sumoylation sites in this process.

Next, we investigated whether the SUMO-modified pool of DRIL1 adopts a particular subnuclear distribution pattern. To characterize the distribution of SUMO1- conjugated DRIL1 in the nucleus, we applied biochemical fractionation of 293T cells transfected with wt DRIL1, K398R or K4xR mutants. Soluble fractions were isolated, and the pellets were treated with DNase I to release chromatin-bound proteins. Then, nuclear matrix proteins were extracted in high salt buffer (see materials and methods). These fractions will be referred to as ‘soluble’, ‘chromatin’, and ‘MAR’ fraction, respectively. Equivalent proportions of these three fractions were probed with DRIL1 antibody by Western immunoblotting. Controls for soluble (tubulin), chromatin (histone H1), and nuclear matrix (lamin B) fractions revealed no cross-contamination (Fig. 3B, lower panels). SUMO1 conjugated DRIL1 was solubilized only by the last high salt treatment (Fig. 3B, upper panel), indicating that SUMO1 conjugated DRIL1 is exclusively present in the MAR fraction. Since PIASy is also a MAR protein and acts as a SUMO E3 ligase for DRIL1, this raises the possibility that sumoylation of DRIL1 takes place in the MARs. From these results, we conclude that sumoylation does not play a crucial role in the nucleocytoplasmic trafficking of DRIL1. Rather, sumoylation of DRIL1 may modulate MAR-specific DRIL1 function.

### Sumoylation does not affect DRIL1 DNA-binding activity

**Figure 4:**
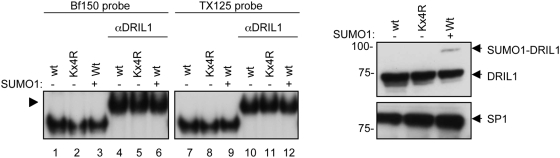
Sumoylation does not affect DRIL1 DNA-binding activity. Nuclear extracts from 293T cells transiently transfected with wt DRIL1, K398R or Kx4R and SUMO1 were prepared. EMSA was performed using ^32^P-labeled, Bf150 and TX125 prototypic MAR probes (left panel). Supershift was performed with anti-DRIL1 antibody (arrow indicate supershifted DRIL1 complex). Equivalent input was confirmed by Western blotting, using antibodies against DRIL1 and SP1 as loading control (right panel).

DRIL1 displays affinity towards A/T DNA sequences through its AT-rich interaction domain (ARID). To determine whether DRIL1 sumoylation modulates this interaction, we performed an electromobility shift assay (EMSA) to examine the binding of wild-type and non-sumoylable DRIL1 to two oligonucleotides representing prototypic DRIL1 binding sites (bf150 and TX125, respectively [6]). For both probes, specific DNA binding activity, as confirmed by a DRIL1 antibody supershift, was detected in samples containing equal amounts of both exogenous wild-type and SUMO-defective DRIL1 (Fig. 4). Furthermore, DRIL1 DNA binding activity was unaffected by co-expression of SUMO1 (Fig. 4, lane 3 and 9). These results suggest that the sumoylated species of DRIL1 do not contribute to the total DRIL1-binding activity in a major way.

### Sumoylation of DRIL1 impairs its interaction with E2F1

**Figure 5:**
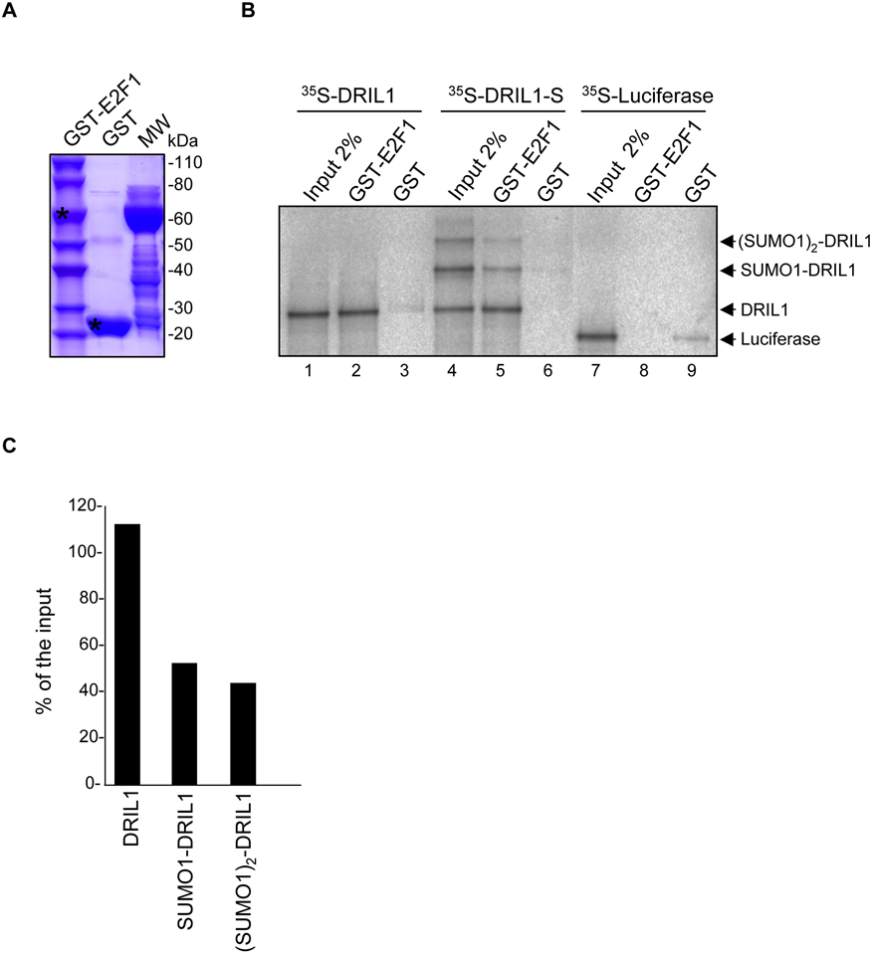
Sumoylation impairs interaction of DRIL1 with E2F1 *in vitro*. (A) Coomassie staining of the gel showing expression of recombinant GST-fusion proteins purified from bacteria, asterix for full length recombinant GST-E2F1 and GST. (B) GST pull-down assay of *in vitro* translated unsumoylated (DRIL1) or sumoylated (DRIL1-S) DRIL1, and Luciferase control, with GST-E2F1 and GST control. (C) Quantification by ImageJ software of DRIL1 species recovered by pulldown vs input (% of input band intensity).

DRIL1 binds to E2F1 both *in vivo* and *in vitro*, and exerts cooperativity towards E2F-dependent transcription [1]. Because DRIL1 interaction with E2F1 involves a region including the SUMO target lysine K398, we hypothesized that sumoylation might influence DRIL1-E2F1 binding. Therefore, we tested whether SUMO-modified DRIL1 can still bind directly to E2F1. For this, we performed an *in vitro* binding assay using an unmodified or a sumoylated ^35^S-labeled DRIL1 with recombinant GST-E2F1 or GST protein as a control (Fig. 5A). We first confirmed the binding of E2F1 to DRIL1 (Fig. 5B, lane 2). However, when a mixture containing comparable amount of unsumoylated and monosumoylated DRIL1 (Fig. 5B, lane 4) was assayed for binding to E2F1, we observed that E2F1 preferentially interacts with unsumoylated DRIL1 (Fig. 5B, lane 5). Quantification of E2F1-bound species further revealed a decrease of more than 50% of sumoylated DRIL1, compared to the input, while conversely a 12% increase is observed for unsumoylated DRIL1 (Fig. 5C). These results suggest that sumoylation of DRIL1 impairs its interaction with E2F1. However, we do observe some residual binding of sumoylated DRIL1 to E2F1, this, could possibly be indirect and due to DRIL1/SUMO1-DRIL1 heterodimerisation, as DRIL1 was previously shown to self-associate through its REKLES subdomain [12].

Supporting this hypothesis, fully sumoylated DRIL1 species, obtained by *in vitro* sumoylation in presence of PIASy, do not interact with E2F1, unless unsumoylated DRIL1 is added to the binding reaction (data not shown). Regardless, these results show that DRIL1 sumoylation is a key regulator of the interaction between DRIL1 and E2F1.

### Sumoylation modifies DRIL1 transcriptional activity

Sumoylation can have an inhibitory role in transcription by leading to the recruitment of repressive factors with chromatin remodeling activity or by initiating the formation of repressive complex [18]. In order to test whether SUMO modification of DRIL1 can modulate its transcriptional activity, we expressed wt, K398R, Kx4R mutants or empty vector in 293T cells and performed genome-wide expression microarray analysis (the raw data of the microarray analyses are available at http://www.ebi.ac.uk/microarray-as/aer/#ae-main[0], accession E-TABM-681). We found 215 genes significantly regulated by wt DRIL1 when compared to empty vector (p<0.01), of which 86% were upregulated (table S1). Among those genes, 47 were regulated by wt DRIL1 only, pointing to a possible SUMO-specific regulation of this subset of genes (table S1, bold).

**Figure 6:**
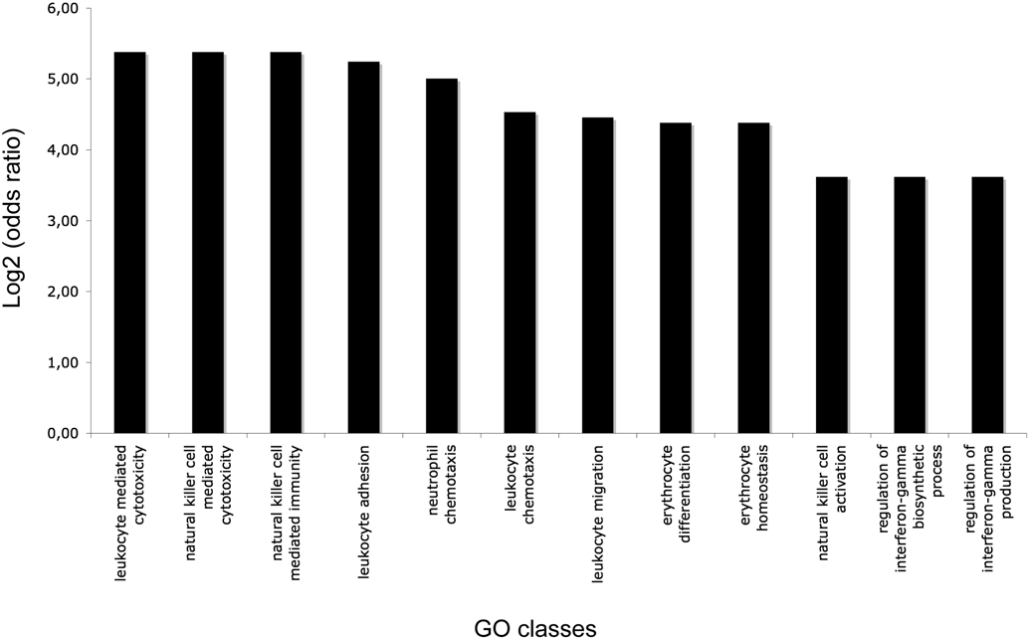
DRIL1 sumoylation controls leukocyte biological processes. Unbiased Gene ontology (GO) analysis was applied on the DRIL1 microarray data sets and 12 out of 86 significantly upregulated classes by wt DRIL1 only (those that are implicated in leukocyte biological processes) are represented in the chart (the full panel is represented in Table S2). Log2 (odds ratio) of the enrichment of the GOID were obtained using Gene Ontology Enrichment Analysis Software Toolkit ([19], http://omicslab.genetics.ac.cn/GOEAST/).

To test whether SUMO modification of DRIL1 leads to regulation of a subset of genes implicated in a specific biological process, we performed an unbiased Gene Ontology (GO) analysis using Gene Ontology Enrichment Analysis Software Toolkit ([19], http://omicslab.genetics.ac.cn/GOEAST/). We found 193 GO classes enriched by wt DRIL1 (table S2), among them 86 GO classes were not found in the SUMO mutants (table S2, bold), suggesting a SUMO-specific regulation of these GO classes by DRIL1. Interestingly, among the enriched GO classes by wt DRIL1 only, we found 12 GO classes implicated in the control of multiple biological processes of leukocytes, including leukocyte migration, differentiation and activation (figure 6). Non of the GO classes implicated in leukocytes processes were enriched by the SUMO mutants. Whether this contributes to specific functions of DRIL1 in leukocytes fate needs further study. This not withstanding, these results reveal an unexpected role of SUMO-modification of DRIL1 in the control of a specific subset of genes implicated in immune cell types regulation.

## Discussion

DRIL1 was previously described to interact with Ubc9, the only known SUMO E2 conjugating enzyme [14]. In this study, we demonstrate that DRIL1 is sumoylated both *in vitro* and *in vivo*, and identified the corresponding site, lysine 398. Interestingly, it was recently found that RBP1, another member of the ARID DNA-binding family protein, is also SUMO-modified [16]. RBP1 sumoylation occurs in two SUMO acceptor lysine residues within the transcriptional repression region R1 of the protein [16]. Mutation of these specific lysines leads to an inhibition of the repression activity of the R1 region. Importantly, among the ARID family proteins, ARID3C and RBP1 share with DRIL1 the same IKKE sumo site, revealing a putative conserved SUMO motif in a subset of ARID family members. Our search for SUMO motifs within the 15 human ARID family proteins using SUMOplot™ analysis program led us to find that all the ARID members harbor multiple putative SUMO sites (data not shown). Altogether, these data might pinpoint a common post-translational modification that could regulate the activity of the entire ARID family proteins.

SUMO E3 ligases have been shown to considerably enhance the sumoylation efficiency by bridging SUMO-Ubc9 to specific target proteins [12]. In this study, we found that the SUMO E3 ligase PIASy is able to catalyse the SUMO modification of DRIL1 *in vitro* and *in vivo*. Interestingly, PIASy is the only matrix-associated SUMO E3 ligase that was described so far [20]. These results are particularly interesting because SUMO1 conjugated DRIL1 is exclusively located in the MAR fraction, suggesting that endogenous DRIL1 might be SUMO modified on this nuclear fraction.

DRIL1 is a nuclear protein that contains a putative nuclear localization signal (NLS) KIKK [21]. Although K398 is the central lysine of this putative NLS sequence, the SUMO DRIL1 mutant K398R was not mislocalized (figure 3). These results confirmed previous observations made by Nixon et al., who showed that a KIKK BRIGHT deletion mutant was not excluded from the nucleus [21], revealing that BRIGHT enters the nucleus through other means. These data were confirmed recently also by Philip Tucker's group showing that BRIGHT was able to shuttle from the cytoplasm to the nucleus via a complex mechanism involving a non-classical NLS and a nuclear export signal (NES) both located in the REKLES domain that are regulated by other functional domains of BRIGHT including the N-terminal acidic region and a large central region including the KIKK motif [17], [22]. Collectively, these results show that the SUMO modification of DRIL1 in K398 might be necessary but not sufficient for the nuclear localization or the shuttling.

We recently demonstrated that DRIL1 allows primary mouse fibroblasts (MEFs) to efficiently bypass both spontaneous and activated RAS-induced senescence by deregulating the Rb/E2F1 pathway [10]. We tested whether the SUMO modification of DRIL1 was implicated in this phenomenom. Like wt DRIL1, all the SUMO mutants were able to bypass both spontaneous and activated RAS-induced senescence (data not shown), reaveling that this modification is not implicated in this process.

DRIL1 was first described as an E2F1-binding protein that stimulates E2F-dependent transcription by forming heterodimers. DRIL1 interacts with E2F1 through a large domain that include IKKE and REKLES motifs [1]. We show in our study that DRIL1 sumoylation impairs the interaction between DRIL1 and E2F1 *in vitro* (figure 5). Further investigation will be needed to elucidate the precise role of DRIL1 sumoylation on E2F1 activity *in vivo*. The fact that ARID3C and RBP1 share with DRIL1 the IKKE sumoylation site might suggest that they are putative E2F1 interacting proteins.

The ARID DNA-binding family protein exhibits a range of cellular functions, including chromatin remodeling and regulation of gene expression during cell growth, differentiation and development [23]. This study is the first to analyze the DRIL1 transcriptome in a genome-wide fashion. We found 215 genes that are significantly modulated by DRIL1 and in most of the cases we observed an upregulation of the target genes (table S1). Among them, 47 genes were specifically modulated by wt DRIL1 and not by any of the SUMO mutants. We performed a GO class analysis and found a SUMO-dependent enrichment of genes implicated in leukocyte lineages (figure 6). This result reveals a crucial role for SUMO modification of DRIL1 in the control of its transcriptional activity. Indeed, DRIL1 was first described as a regulator of IgH expression [6] and our data are now showing that DRIL1 might also control the expression of other key genes implicated in the differentiation and activation of lymphocytes. In particularly, C/EBPγ (a transcription factor that activates the transcription of the interleukin-6 and interleukin-8 in B lymphoblast cells; [24]), integrin β2 (CD18, a key cell surface adhesion receptor, expressed in leukocytes and required for the recruitment and the activation of polymorphonuclear neutrophils during inflammation; [25]) and UL16 binding protein 1 (ULBP1, ligand for NKG2D receptor that activate multiple signaling pathways in primary NK cells, resulting in the production of cytokines and chemokines; [26]). Further experiments will be needed to confirm that these genes are direct target genes and validate these targets in other cell systems, particularly lymphocytes.

Interestingly, Siatecka and co-workers have obtained similar results with the transcription factor Erythroid Kruppel-like factor (EKLF) [27]. Indeed, sumoylation of EKLF promotes transcriptional repression and is involved in inhibition of megakaryopoiesis. These results may shed light on a key role of sumoylation in the control of transcription factors implicated in lineage decision.

In conclusion, we have identified a new post-translational modification of DRIL1 that constitute an important mechanism for the tight regulation of the activity of this transcription factor and in the control of leukocyte fate.

## Materials and Methods

### Plasmids and antibodies

pcDNA3.1-wtDRIL1 construct was already described [10]. DRIL1 SUMO mutations were obtained by site-directed mutagenesis using the Quick-Change Site Directed Mutagenesis kit (Stratagene). pSG5T7-PIAS1, pSG5T7-PIAS3, pSG5T7-PIASXalpha, pSG5T7-PIASXbeta, pCDNA3.1-SUMO1 and pCDNA3.1-SUMO2 constructs were a kind gift from Dr. A. Dejean (Institut Pasteur, France).

Immunoblotting was performed with the following antibodies: rabbit DRIL1 and mouse T7 from Bethyl laboratory, mouse SUMO1 and goat lamin B from Santa-cruz, mouse β-actin and mouse α-tubulin from Sigma, mouse Histone H1 from Roche, mouse SP1 from Abcam. Conjugated with horseradish peroxidase goat anti-mouse from BioRad, goat anti-rabbit and swine anti-goat from Biosource.

### Cell culture and Transfections

293T cells were cultured in DMEM (Life technologies) supplemented with 10% FCS (Greiner bio-one), 2 mM L-glutamine, 100 units.ml^−1^ penicillin and 0.1 mg.ml^−1^ streptomycin (all Gibco).

293T cells (10^6^) were plated in 10 cm dishes 12 hours before transfection. The cells were transiently transfected using standard calcium phosphate transfection [28]. For co-transfection, we equalized the total DNA amounts by adding the appropriate amount of pCDNA3.1 empty circular vector (Invitrogen).

### *In vitro* Transcription/Translation and *in vitro* sumoylation assay

Wt DRIL1, SUMO mutants DRIL1 and all PIAS were expressed using the TNT Coupled reticulocyte lysate system (Promega) following the manufacter's instructions, in the presence or not of ^35^S-Methionine (Promix, Amersham). Protein integrity and level were assessed by SDS-PAGE. The recombinant SUMO1, Ubc9, Aos1/Uba2 and RanBP2ΔFG were a kind gift from Dr. T. Sixma (NKI, The Netherlands).

The sumoylation reaction was carried out as follows in a total volume of 20 µl at 32°C for 1 hour (unless otherwise stated): 3 µl of ^35^S-Methionine labeled *in vitro* translated target protein, 10 µM SUMO1, 200 nM Aos1/Uba2, 700 nM Ubc9 recombinant proteins, final reaction buffer (20 mM HEPES, 5 mM MgCl2, 2 mM KAc, 0.1 mg/ml BSA, 0.02% Tween 20; 0.05 mM DTT, 5 mM ATP, ddH2O). The reactions were analyzed by autoradiography. For the E3 ligase assay, 1 µl of unlabeled *in vitro* translated PIAS proteins or 5 nM final of recombinant RanBP2ΔFG fragment were added to the SUMO reaction.

### Western blotting and immunoprecipitations

Cells were washed with PBS and lysed in RIPA buffer (1% NP40, 1% SDS, 0.5 DOC, 50 mM TRIS-HCl pH 8, 2 mM EDTA) supplemented with N-ethylameinide (NEM, Sigma), protease inhibitor cocktail (Roche) and phosphatase inhibitor (Sigma) After measuring concentrations using Bradford method (Biorad) equal amounts of protein were loaded onto precast gradients gels (NuPage, Invitrogen) and subjected to SDS-PAGE using standard procedures. Samples were transferred to nitrocellulose membranes (Millipore). We used secondary antibodies conjugated with horseradish peroxidase, and developed the blots using ECL (Dura, Pierce).

293T cells were tansfected with pcDNA3.1 wt DRIL1, grown for 48 hours in a 6-well plate and lysed in 0.3 ml of lysis buffer (50 mM HEPES-KOH, pH 7.4; 200 mM KCl; 10% glycerol; 1% Nonidet P-40; 1 mM EDTA, and 1 mM DTT) supplemented with 0.3 µl of protease inhibitor mixture (Roche) and incubated on ice for 5 minutes. After centrifugation at 12,000 rpm for 15 minutes, the supernatant was incubated with 40 µl of 50% protein-A-Sepharose beads (Amersham Biosciences) with constant agitation for 30 min. The supernatant was transferred to a fresh 1.5-ml tube after centrifugation at 2,000 rpm for 1 minute and 30 µl of the supernatant was saved for the input samples. Three µl of anti-DRIL1 antibody was incubated with the lysate for 1 hour with constant agitation. Then 30 µl of 50% protein-A-Sepharose beads was added to the lysate and incubated for 2 hours as above. Beads were washed three times by rounds of agitation (in 1 ml of lysis buffer for 5 minutes) and centrifugation. After the final wash, the beads were suspended in 37.5 µl of 1×SDS sample buffer and boiled for 5 minutes. The input samples were mixed with 7.5 µl of 5×SDS sample buffer and boiled for 5 minutes. Lysates were subjected to SDS-PAGE as described above and western blotted with antibodies against DRIL1 and SUMO1.

### Immunofluoresence

Twenty-four hours after transfection, 10^5^ cells were plated on LabTek slides (Nalge Nunc International) and left overnight in complete medium, washed in PBS, fixed in 4% PBS-buffered formaldehyde for 15 minutes, permeabilized in 0.2% tritonX-PBS, blocked in PBS-buffered 5% normal goat serum, 0.2% tween20, 0.2% gelatin for 1 hour, incubated with DRIL1 antibody (1∶200) for 45 minutes, incubated with Alexa Fuor 568 (1∶200) secondary antibody (Invitrogen) for 30 minutes and counterstained with TO-PRO-3 (Invitrogen).

### Nuclear protein extraction and subnuclear fractionation into Nuclear Matrix

For nuclear protein extraction, 10^7^ cells were washed and scrapped in ice-cold PBS. The pellet was washed in 500 µl wash buffer (10 mM HEPES pH 7.9, 20 mM KCl, 2 mM MgCl2, 0.1 mM EDTA pH 7), centrifuged 1 minute, lysed in 300 µl of cytoplasmic extraction buffer (10 mM HEPES pH 7.9, 10 mM KCl, 2 mM MgCl2, 0.1 mM EDTA pH 7, 0.2% NP40) supplemented with protease inhibitor cocktail and centrifuged to removed cellular debris. Pelleted nuclei were resuspended in 110 µl nuclear extract buffer (20 mM HEPES pH 7.9, 0.63 M NaCl, 1.5 mM MgCl2, 0.2 mM EDTA pH 7, 25% glycerol) supplemented with protease inhibitor cocktail, incubated for 30 minutes at 4°C, centrifuged for 30 minutes at 14,000 RPM and the protein amounts were quantified using Bradford method.

Fractionations were preformed according to the method of Reyes et al. [29]. Briefly, following transfection with wt and mutant constructs, 293T cells (in 6-well plates) were scraped, washed, and resuspended in 80 µl of CSKT (10 mM PIPES, pH 6.8; 100 mM NaCl; 300 mM sucrose; 3 mM MgCl2; 1 mM EGTA; 1 mM DTT, and 0.5% Triton X-100 supplemented with 0.5 µl of protease inhibitor mixture). The suspension was incubated for 5 minutes and centrifuged at 7,500 rpm for 3 minutes. The supernatant was used for the soluble fraction, and the pellet was washed with CSK (CSKT minus Triton X-100), centrifuged, and suspended in 60 µl of CSK supplemented with 6 µl of RNase-free DNase I (Invitrogen). The suspension was incubated at 37°C for 2 hours and then 20 µl of 1 M ammonium sulfate (NH4)2SO4/CSK was added to a final concentration of 0.25 M (NH4)2SO4. After incubation at 4°C for 5 minutes, the suspension was centrifuged at 7,500 rpm for 3 minutes. The supernatant contained the chromatin fraction. The pellet, containing the nuclear matrix fraction, was suspended in 80 µl of 2 M NaCl/CSK and centrifuged again at 7,500 rpm for 3 minutes. The pellet was suspended in 80 µl of 8 M urea buffer (8 M urea and 10 mM Tris-Cl, pH 8.0).

### ElectroMobility Shift Assay (EMSA)

Binding reactions were performed for 30 min at room temperature with 5 µg nuclear proteins in 20 mM HEPES, pH 7.9, 10 mM KCl, 0.2 mM EDTA, 20% (v/v) glycerol, 1% (wt/v) acetylated BSA, 3 µg of poly(dI-dC) (Amersham Pharmacia Biotech, Aylesbury, U.K.), 1 mM DTT, 1 mM PMSF, and 100,000 cpm of ^32^P-labeled double-stranded oligonucleotide probes. Probes were prepared by annealing the appropriate single-stranded oligonucleotide (Sigma) at 65°C for 10 minutes in 10 mM Tris, 1 mM EDTA, 10 mM NaCl, followed by slow cooling to room temperature. The probes were then labeled by end-filling with the Klenow fragment of Escherichia coli DNA polymerase I (Roche), with [{alpha}-32P]-dATP and [{alpha}-32P]-dCTP (Promix, Amersham). Labeled probes were purified by spin chromatography on Sephadex G-25 columns (Roche). DNA-protein complexes were separated from unbound probe on 4% native polyacrylamide gels at 150 V in 0.25 M Tris, 0.25 M sodium borate, and 0.5 mM EDTA, pH 8.0. Gels were vacuum-dried and exposed to Kodak x-ray film at −80°C for 12 hours. For supershifting experiments, 1.5 µl of DRIL1 antibody was incubated with the extracts for 30 min before addition of the radiolabeled probe. The sequences of the oligonucleotide used in this work were as follows: palindromic Bf150 probe, 5′- GGTTCCCCAAAATATAAGTATAAATATGTGCAAAACTTGTTTATTAACTTATTTATCTTAAAATCTGG-3′; palindromic TX125 probe, 5′- GGTTAACTTGTTAAATCACAATAAAATATTGAAGTGTTATCACATACACATACTAAACAATTTTCTAA-3′.

### GST *in vitro* binding assay

GST-E2F1 and GST proteins were expressed in E. coli BL21 for 4 hours at 37°C by induction with 0.2 mM IPTG (Sigma). After bacterial lysis, recombinant proteins were purified on glutathione-Sepharose beads (Amersham). 20 µl of ^35^S-Methionine-labeled *in vitro* translated DRIL1, unmodified or sumoylated, was incubated with GST-E2F1 or GST proteins loaded on glutathione-Sepharose 4b beads for 2 hours at 4°C in GST binding buffer (50 mM Tris pH 7.5, 150 mM NaCl, 0.1% Nonidet P-40, 1 mM DTT, 20 mM NEM, 1X protease inhibitors cocktail tablets, Roche). The input samples were incubated for the same duration and temperature as the binding reactions to enable direct comparison. Beads were washed five times with binding buffer, and bound proteins were eluted with 2X SDS sample buffer and analyzed by gel electrophoresis, followed by autoradiography.

### Microarray gene expression analysis

Total RNA was isolated by using RNeasy Protect Mini Kit (Qiagen) according to the manufacturer instruction. 350 ng of total RNA was reverse transcribed and amplified overnight with T7 RNA polymerase and labeled with biotin following the manufacturer's protocol. 1.5 microgram of biotin-labeled cRNA was hybridized to Illumina Human WG-6 BeadChips at 55°C for 18 hrs. BeadChips were incubated with Cy3 streptavidin and washed according to the manufacturer's protocol. The hybridized BeadChips were scanned by Illumina BeadScan confocal scanner and analyzed by Illumina's BeadStudio version 1.5.1.3. Data were normalized with the Illumina Beadstudio software, using average normalization.

## Supporting Information

Table S1 List of genes modulated by wt DRIL1. Mean average signal for microarrays (AVG_signal) from two independent experiments. Genes significantly modulated in the two independent experiments (p<0.01 for each microarray). (-) for 293T infected by empty vector, wt for 293T infected by wt DRIL1. In bold, genes modulated by wt DRIL1 only.
(0.07 MB PDF)

Table S2 List of Gene Ontology (GO) classes enriched by wt DRIL1. Log2 (odds ratio) of the enrichment of the GOID were obtained using Gene Ontology Enrichment Analysis Software Toolkit ([19], http://omicslab.genetics.ac.cn/GOEAST/). P-value of the significance for the enrichment in the dataset of the listed GOID using multiple-test adjusted false discovery rate (FDR). In bold, GO classes enriched by wt DRIL1 only.
(0.05 MB PDF)
