# Supplementary material for: SUMOylation of DRIL1 Directs Its Transcriptional Activity Towards Leukocyte Lineage-Specific Genes
Source: PLoS One. 2009 May 14;4(5):e5542. doi: 10.1371/journal.pone.0005542 (PMC2677661; doi:10.1371/journal.pone.0005542)
Supplement: Table S1 — List of genes modulated by wt DRIL1. Mean average signal for microarrays (AVG_signal) from two independent experiments. Genes significantly modulated in the two independent experiments (p<0.01 for each microarray). (-) for 293T infected by empty vector, wt for 293T infected by wt DRIL1. In bold, genes modulated by wt DRIL1 only. (0.07 MB PDF) [file pone.0005542.s001.pdf]

Gene ID	Definition	REFSEQ ID	(-) AVG_signal	wt AVG_signal	Fold change
BGN	biglycan	NM_001711.3	34.04	257.39	7.56
GDF15	growth differentiation factor 15	NM_004864.1	41.41	258.91	6.25
HES5	hairy and enhancer of split 5	NM_001010926.1	60.29	319.40	5.3
H19	H19 imprinted maternally expressed untranslated	NR_002196.1	37.31	187.94	5.04
MYBPHL	myosin binding protein H-like	NM_001010985.1	38.12	181.96	4.77
CELP	carboxyl ester lipase pseudogene	NR_001275.1	35.70	151.16	4.23
CDKN1A	cyclin-dependent kinase inhibitor 1A	NM_078467.1	301.82	1223.12	4.05
CEL	carboxyl ester lipase	NM_001807.2	57.66	227.88	3.95
CRIP2	cysteine-rich protein 2	NM_001312.2	48.45	177.93	3.67
GPR56	G protein-coupled receptor 56	NM_201525.1	36.22	132.45	3.66
ANKRD37	ankyrin repeat domain 37	NM_181726.1	67.79	234.27	3.46
FLJ13841	hypothetical protein LOC79755	NM_024702.1	39.05	128.17	3.28
**LY6H**	**PREDICTED: lymphocyte antigen 6 complex locus H**	**NM_002347.2**	**37.86**	**121.04**	**3.20**
GADD45A	growth arrest and DNA-damage-inducible alpha	NM_001924.2	231.04	738.72	3.20
INPP5D	inositol polyphosphate-5-phosphatase	NM_001017915.1	38.01	119.34	3.14
ACTA2	actin alpha 2 smooth muscle aorta	NM_001613.1	593.66	1845.84	3.11
CRABP2	cellular retinoic acid binding protein 2	NM_001878.2	139.68	429.74	3.08
MGC59937	Similar to RIKEN cDNA 2310002J15 gene	NM_199001.1	97.96	294.55	3.01
**HS.554203**	**clone FP7915 unknown**		**38.91**	**113.49**	**2.92**
ISG20	interferon stimulated exonuclease gene	NM_002201.4	63.58	184.71	2.90
**ITGB2**	**integrin beta 2**	**NM_000211.2**	**46.19**	**134.14**	**2.90**
ATF3	activating transcription factor 3	NM_001040619.1	360.23	1025.53	2.85
H1F0	H1 histone family member 0	NM_005318.2	1196.86	3392.80	2.83
**LCE1B**	**late cornified envelope 1B**	**NM_178349.1**	**35.33**	**99.35**	**2.81**
PGF	placental growth factor vascular endothelial growth factor-related protein	NM_002632.4	58.37	158.11	2.71
G0S2	G0/G1switch 2	NM_015714.2	40.90	109.88	2.69
ZNF467	zinc finger protein 467	NM_207336.1	72.69	193.06	2.66
RHOB	ras homolog gene family member B	NM_004040.2	425.66	1128.44	2.65
CDKN1C	cyclin-dependent kinase inhibitor 1C	NM_000076.1	50.49	132.44	2.62
UNQ2541	MSFL2541	NM_203347.1	78.65	206.13	2.62
APOE	apolipoprotein E	NM_000041.2	128.49	335.78	2.61
TP53I3	tumor protein p53 inducible protein 3	NM_147184.1	68.99	178.49	2.59
LGALS1	lectin galactoside-binding soluble 1	NM_002305.2	120.58	310.87	2.58
BASP1	brain abundant membrane attached signal protein 1	NM_006317.3	527.04	1349.91	2.56
**INHBE**	**inhibin beta E**	**NM_031479.3**	**48.50**	**123.90**	**2.55**
P4HA1	procollagen-proline 2-oxoglutarate 4-dioxygenase (proline 4-hydroxylase)	NM_000917.2	297.79	756.36	2.54
FEZ1	fasciculation and elongation protein zeta 1	NM_005103.3	182.57	462.59	2.53
IGFBP6	insulin-like growth factor binding protein 6	NM_002178.2	51.98	128.49	2.47
KREMEN2	kringle containing transmembrane protein 2	NM_024507.2	76.75	189.36	2.47
CD24	CD24 molecule	NM_013230.2	109.18	263.37	2.41
BHLHB2	basic helix-loop-helix domain containing class B 2	NM_003670.1	412.91	985.84	2.39
**OLFM1**	**olfactomedin 1**	**NM_014279.2**	**54.04**	**128.25**	**2.37**
SIRT5	sirtuin 5	NM_012241.2	275.10	642.98	2.34
AXUD1	AXIN1 up-regulated 1	NM_033027.2	145.94	341.06	2.35
MGC20983	hypothetical protein MGC20983	NM_145045.3	75.07	175.34	2.34
TNFRSF12A	tumor necrosis factor receptor superfamily member 12A	NM_016639.1	93.83	219.11	2.34
EEF1A2	eukaryotic translation elongation factor 1 alpha 2	NM_001958.2	1262.68	2935.02	2.32
ADM	adrenomedullin	NM_001124.1	254.30	590.72	2.32
TMSL3	thymosin-like 3	NM_183049.2	1161.71	2675.85	2.30
**CYGB**	**cytoglobin**	**NM_134268.3**	**36.68**	**84.22**	**2.30**
CNTFR	ciliary neurotrophic factor receptor	NM_147164.1	136.93	312.89	2.28
**COX11**	**COX11 homolog cytochrome c oxidase assembly protein**	**NM_004375.2**	**129.18**	**292.99**	**2.27**
HIST2H2AA3	histone cluster 2 H2aa3	NM_003516.2	225.47	511.27	2.27
VLDLR	very low density lipoprotein receptor	NM_001018056.1	72.09	163.05	2.26
MT1F	metallothionein 1F	NM_005949.1	145.40	328.83	2.26
**CRLF1**	**cytokine receptor-like factor 1**	**NM_004750.2**	**48.21**	**108.67**	**2.25**
**HS.537451**	**AGENCOURT_7971794 NIH_MGC_67 cDNA clone**		**43.69**	**98.31**	**2.25**
ARRDC4	arrestin domain containing 4	NM_183376.1	156.19	351.42	2.25
HBA1	hemoglobin alpha 1	NM_000558.3	84.73	190.23	2.25
**MDK**	**midkine**	**NM_001012334.1**	**2074.27**	**4653.24**	**2.24**
SLC12A4	solute carrier family 12 (potassium/chloride transporters) member 4	NM_005072.3	57.17	127.83	2.24
PMAIP1	phorbol-12-myristate-13-acetate-induced protein 1	NM_021127.1	228.71	511.36	2.24
SLC2A3	solute carrier family 2 (facilitated glucose transporter) member 3	NM_006931.1	158.04	352.66	2.23
APLP1	amyloid beta (A4) precursor-like protein 1	NM_005166.3	96.47	214.53	2.22
DRD1IP	dopamine receptor D1 interacting protein	NM_015722.2	128.50	285.73	2.22
ALOX5	arachidonate 5-lipoxygenase	NM_000698.2	62.10	137.84	2.22
TXNIP	thioredoxin interacting protein	NM_006472.1	117.04	259.32	2.22
MGC33212	hypothetical protein MGC33212	NM_152773.2	564.41	1248.32	2.21
LOC387763	PREDICTED: hypothetical LOC387763	XM_373497.4	178.15	392.94	2.21
**ULBP1**	**UL16 binding protein 1**	**NM_025218.2**	**51.36**	**113.15**	**2.20**
**TCP10L**	**t-complex 10 (mouse)-like**	**NM_144659.3**	**76.53**	**167.01**	**2.18**
**C1QTNF1**	**C1q and tumor necrosis factor related protein 1**	**NM_198594.1**	**69.45**	**151.05**	**2.17**
S100A10	S100 calcium binding protein A10	NM_002966.2	1999.58	4322,00	2.16
CAV1	caveolin 1 caveolae protein	NM_001753.3	110.37	238.53	2.16
**C10ORF10**	**chromosome 10 open reading frame 10**	**NM_007021.2**	**75.29**	**162.30**	**2.16**
**EPHA2**	**EPH receptor A2**	**NM_004431.2**	**53.76**	**115.84**	**2.15**
RRAS	related RAS viral (r-ras) oncogene homolog	NM_006270.2	114.90	247.44	2.15
PNCK	pregnancy upregulated non-ubiquitously expressed CaM kinase	NM_198452.1	148.79	320.17	2.15
**NPY**	**neuropeptide Y**	**NM_000905.2**	**56.67**	**121.36**	**2.14**
MYOM2	myomesin (M-protein) 2	NM_003970.1	93.50	199.85	2.14
**ZNF654**	**zinc finger protein 654**	**NM_018293.2**	**80.47**	**171.91**	**2.14**
SEMA3F	sema domain immunoglobulin domain (Ig) short basic domain secreted 3F	NM_004186.2	67.08	143.13	2.13

C11ORF54	chromosome 11 open reading frame 54	NM_014039.2	216.51	458.43	2.12
TIP39	tuberoinfundibular 39 residue protein	NM_178449.2	45.56	96.42	2.12
FBLN1	fibulin 1	NM_006486.2	312.72	661.20	2.11
GPRC5C	G protein-coupled receptor family C group 5 member C	NM_018653.3	104.74	221.32	2.11
SNAP91	synaptosomal-associated protein	NM_014841.1	144.19	304.14	2.11
CHRNA3	cholinergic receptor nicotinic alpha 3	NM_000743.2	67.43	141.70	2.10
ENO2	enolase 2	NM_001975.2	159.48	334.91	2.10
BTG2	BTG family member 2	NM_006763.2	113.82	239,00	2.10
NEFM	neurofilament medium polypeptide	NM_005382.1	695.61	1448.62	2.08
**LOC644743**	**PREDICTED: hypothetical protein LOC644743**	**XM_932346.1**	**78.46**	**163,00**	**2.08**
**CTH**	**cystathionase (cystathionine gamma-lyase)**	**NM_153742.3**	**135.69**	**279.11**	**2.08**
CCDC15	coiled-coil domain containing 15	NM_025004.1	93.93	193.17	2.06
ANTXR2	anthrax toxin receptor 2	NM_058172.3	92.60	190.28	2.05
**SYT11**	**synaptotagmin XI**	**NM_152280.2**	**72.73**	**148.93**	**2.05**
TJP3	tight junction protein 3	NM_014428.1	65.61	134.12	2.04
PRPH	peripherin	NM_006262.3	92.91	189.07	2.04
SPATA18	spermatogenesis associated 18 homolog	NM_145263.1	101.25	205.95	2.03
PPM1M	protein phosphatase 1M (PP2C domain containing)	NM_144641.1	174.11	353.27	2.03
RDM1	RAD52 motif 1	NM_001034836.1	378.90	767.51	2.03
CRMP1	collapsin response mediator protein 1	NM_001014809.1	126.13	255.06	2.02
TMEM116	transmembrane protein 116	NM_138341.1	128.82	260.47	2.02
DUSP1	dual specificity phosphatase 1	NM_004417.2	306.82	618.61	2.02
TICAM2	toll-like receptor adaptor molecule 2	NM_021649.3	91.80	184.52	2.01
**PPM1D**	**protein phosphatase 1D magnesium-dependent delta isoform**	**NM_003620.2**	**267.37**	**537.23**	**2.01**
TERF1	telomeric repeat binding factor (NIMA-interacting)	NM_017489.1	266.29	533.49	2,00
DENND2A	DENN/MADD domain containing 2A	NM_015689.2	69.66	139.12	2,00
**RAD51C**	**RAD51 homolog C**	**NM_058216.1**	**657.66**	**1312.6**	**2,00**
AHNAK	AHNAK nucleoprotein	NM_001620.1	134.04	266.90	1.99
**C4ORF34**	**chromosome 4 open reading frame 34**	**NM_174921.1**	**217.83**	**432.13**	**1.98**
ZNF423	zinc finger protein 423	NM_015069.2	91.86	181.98	1.98
**NHLH2**	**nescient helix loop helix 2**	**NM_005599.2**	**59.97**	**118.43**	**1.98**
C1ORF66	chromosome 1 open reading frame 66	NM_015997.2	293.41	577.35	1.97
HSD17B6	hydroxysteroid (17-beta) dehydrogenase 6	NM_003725.2	152.11	299.10	1.97
CRELD1	cysteine-rich with EGF-like domains 1	NM_001031717.1	199.24	390.58	1.96
**SERPINI1**	**serpin peptidase inhibitor clade I (neuroserpin) member 1**	**NM_005025.2**	**82.43**	**161.11**	**1.95**
LOC153222	adult retina protein	NM_153607.1	104.7	204.21	1.95
RPL37	ribosomal protein L37	NM_000997.3	305.41	594.70	1.95
S100A11	S100 calcium binding protein A11	NM_005620.1	136.42	264.46	1.94
C6ORF148	chromosome 6 open reading frame 148	NM_030568.3	76.50	147.77	1.93
KRCC1	lysine-rich coiled-coil 1	NM_016618.1	94.37	181.32	1.92
NPTX2	neuronal pentraxin II	NM_002523.1	256.91	491.70	1.91
CACNB3	calcium channel voltage-dependent beta 3 subunit	NM_000725.2	156.84	299.94	1.91
PRDM16	PR domain containing 16	NM_022114.2	81.20	155.13	1.91
PPP1R13L	protein phosphatase 1 regulatory (inhibitor) subunit 13 like	NM_006663.2	71.76	137.07	1.91
YPEL3	yippee-like 3	NM_031477.3	160.17	304.53	1.90
TSPAN5	tetraspanin 5	NM_005723.2	136.18	258.36	1.90
DPH5	DPH5 homolog (S cerevisiae) (DPH5) transcript variant 3	NM_001077395.1	539.15	1020.25	1.89
NDRG1	N-myc downstream regulated gene 1	NM_006096.2	164.76	311.41	1.89
ZMAT3	zinc finger matrin type 3	NM_152240.1	214.20	404.81	1.89
LOC112703	hypothetical protein BC004941	NM_138411.1	260.27	491.52	1.89
NAV2	neuron navigator 2	NM_145117.3	76.81	144.67	1.89
ASS	argininosuccinate synthetase	NM_000050.3	812.99	1528.94	1.88
FAM46C	family with sequence similarity 46 member C	NM_017709.2	93.46	175.08	1.87
**TMEM31**	**transmembrane protein 31**	**NM_182541.2**	**76.79**	**143.78**	**1.87**
**RHOC**	**ras homolog gene family member C**	**NM_175744.3**	**919.28**	**1717.68**	**1.87**
ELP4	elongation protein 4	NM_019040.2	134.46	250.33	1.86
**OSBPL7**	**oxysterol binding protein-like 7**	**NM_145798.2**	**83.19**	**153.25**	**1.84**
**LOC646463**	**PREDICTED: similar to Ubiquitin-conjugating enzyme E2 H**	**XM_929387.1**	**102.61**	**188.74**	**1.84**
ANKRA2	ankyrin repeat family A (RFXANK-like) 2	NM_023039.2	288.48	528.93	1.83
**YPEL5**	**yippee-like 5**	**NM_016061.1**	**348.24**	**637.23**	**1.83**
CCNB1IP1	cyclin B1 interacting protein 1	NM_182851.1	1159.66	2120.98	1.83
HS.137274	602572519F1 NIH_MGC_77 cDNA clone		123.79	226.39	1.83
IMMP1L	IMP1 inner mitochondrial membrane peptidase-like	NM_144981.1	229.77	419.09	1.82
**DKFZP686K16132**	**similar to BMP2 inducible kinase**	**NM_001012987.1**	**109.44**	**199.52**	**1.82**
**EGR1**	**early growth response 1**	**NM_001964.2**	**85.66**	**155.60**	**1.82**
**THAP10**	**THAP domain containing 10**	**NM_020147.2**	**219.01**	**397.79**	**1.82**
FLJ21839	ATP/GTP binding protein-like 5	NM_021831.5	156.05	283.38	1.82
HERC4	hect domain and RLD 4	NM_015601.2	210.83	381.75	1.81
**RYBP**	**RING1 and YY1 binding protein**	**NM_012234.3**	**264.08**	**473.83**	**1.79**
TMEM54	transmembrane protein 54	NM_033504.2	187.82	336.37	1.79
SFRS14	splicing factor arginine/serine-rich 14	NM_014884.1	547.56	979.11	1.79
**MBNL2**	**muscleblind-like 2**	**NM_207304.1**	**197.37**	**352.93**	**1.79**
**XPC**	**xeroderma pigmentosum complementation group C**	**NM_004628.3**	**638.80**	**1141.58**	**1.79**
AGTPBP1	ATP/GTP binding protein 1	NM_015239.1	252.72	448.25	1.77
C1QL1	complement component 1 q subcomponent-like 1	NM_006688.3	95.87	169.99	1.77
GARNL4	GTPase activating Rap/RanGAP domain-like 4	NM_015085.3	137.01	242.13	1.77
MGC16169	hypothetical protein MGC16169	NM_033115.2	113.30	199.62	1.76
ELMOD1	ELMO/CED-12 domain containing 1	NM_018712.2	137.29	241.67	1.76
LRCH2	leucine-rich repeats and calponin homology (CH) domain containing 2	NM_020871.2	363.50	639.19	1.76
KHDRBS3	KH domain containing RNA binding signal transduction associated 3	NM_006558.1	718.78	1255.37	1.75
DDIT4	DNA-damage-inducible transcript 4	NM_019058.2	2527.63	4409.81	1.74
ALDOC	aldolase C fructose-bisphosphate	NM_005165.2	165.59	288.72	1.74
**LIAS**	**lipoic acid synthetase**	**NM_006859.2**	**181.02**	**314.98**	**1.74**

**CORO2A**	**coronin actin binding protein 2A**	**NM_052820.2**	**187.5**	**326.23**	**1.74**
RBP1	retinol binding protein 1 cellular	NM_002899.2	377.62	656.64	1.74
MIB2	mindbomb homolog 2	NM_080875.1	182.37	314.86	1.73
**MGC15476**	**dapper antagonist of beta-catenin homolog 3**	**NM_145056.1**	**129.64**	**221.14**	**1.71**
HS.371609	cDNA clone IMAGE:5261213		188.47	320.69	1.71
LOC441763	PREDICTED: hypothetical LOC441763	XM_930284.1	702.19	1192.69	1.71
C6ORF48	chromosome 6 open reading frame 48	NM_001040437.1	2048.27	3460.71	1.69
ARHGEF6	Rac/Cdc42 guanine nucleotide exchange factor (GEF) 6	NM_004840.2	346.19	584.10	1.69
ARHGEF2	rho/rac guanine nucleotide exchange factor (GEF) 2	NM_004723.2	474.88	801.07	1.69
**FLJ20130**	**nucleoporin 62kDa C-terminal like**	**NM_017681.1**	**478.84**	**805.42**	**1.68**
**NRIP3**	**nuclear receptor interacting protein 3**	**NM_020645.1**	**220.87**	**370.01**	**1.68**
PPP1R15A	protein phosphatase 1 regulatory (inhibitor) subunit 15A	NM_014330.2	256.35	427.17	1.66
PPP3R1	protein phosphatase 3 (formerly 2B) regulatory subunit B alpha isoform	NM_000945.3	379.54	627.05	1.65
**CEBPG**	**CCAAT/enhancer binding protein (C/EBP) gamma**	**NM_001806.2**	**668.45**	**1103.64**	**1.65**
C12ORF57	chromosome 12 open reading frame 57	NM_138425.2	1297.6	2115.96	1.63
ITPR3	inositol 145-triphosphate receptor type 3	NM_002224.1	451.54	691.98	1.53
**CCDC90B**	**coiled-coil domain containing 90B (CCDC90B)**	**NM_021825.3**	**933.41**	**1420.36**	**1.52**
BPNT1	3'(2') 5'-bisphosphate nucleotidase 1	NM_006085.3	421.30	616.40	1.46
CDC14B	CDC14 cell division cycle 14 homolog B	NM_003671.2	1780.54	2453.87	1.38
LOC644033	PREDICTED: similar to similar to RPL23AP7 protein	XM_927280.1	604.33	373.36	0.62
DHCR24	24-dehydrocholesterol reductase	NM_014762.2	402.73	244.18	0.61
POLR3H	polymerase (RNA) III (DNA directed) polypeptide H	NM_138338.2	561.87	329.44	0.59
**CLUAP1**	**clusterin associated protein 1**	**NM_024793.1**	**18244.89**	**10675.79**	**0.59**
MCOLN2	mucolipin 2	NM_153259.2	517.45	302.33	0.58
RCC1	regulator of chromosome condensation 1	NM_001269.2	253.55	146.82	0.58
MGST1	microsomal glutathione S-transferase 1	NM_145792.1	220.68	125.43	0.57
HS.535028	cDNA: FLJ22720 fis clone HSI14320		439.36	241.80	0.55
SLC25A10	solute carrier family 25 member 10	NM_012140.3	381.45	205.82	0.54
PRPF4B	PRP4 pre- processing factor 4 homolog B	NM_003913.3	261.70	140.53	0.54
ZF	HCF-binding transcription factor Zhangfei	NM_021212.1	358.86	190.41	0.53
**CALML4**	**calmodulin-like 4**	**NM_001031733.1**	**425.23**	**221.28**	**0.52**
CIRBP	cold inducible RNA binding protein	NM_001280.1	3591.26	1864.55	0.52
AK2	adenylate kinase 2	NM_013411.3	356.21	183.94	0.52
LOC442578	similar to Cohesin subunit SA-3	NM_001013739.1	813.28	419.81	0.52
SNORA32	small nucleolar RNA H/ACA box 32	NR_003032.1	169.34	87.28	0.52
LOC645236	PREDICTED: similar to similar to RPL23AP7 protein	XM_928275.1	209.49	107.77	0.51
**DIDO1**	**death inducer-obliterator 1**	**NM_080797.2**	**292.32**	**146.23**	**0.50**
CLK1	CDC-like kinase 1	NM_004071.2	201.92	99.42	0.50
HAGHL	hydroxyacylglutathione hydrolase-like	NM_207112.1	140.01	68.44	0.49
RNU70	small nucleolar RNA H/ACA box 70	NR_000011.1	786.40	378.41	0.48
LOC85390	RNA small nucleolar	NR_001454.1	111.80	52.87	0.47
NSUN5C	NOL1/NOP2/Sun domain family member 5C	NM_149379.1	316.79	147.92	0.47
RNU30	small nucleolar RNA C/D box 30	NR_002561.1	145.84	67.22	0.46
JOSD3	Josephin domain containing 3	NM_024116.2	279.89	127.64	0.46
C1ORF63	chromosome 1 open reading frame 63	NM_207035.1	290.77	130.46	0.45
CSTF3	cleavage stimulation factor 3' pre-RNA subunit 3	NM_001033506.1	298.22	133.26	0.45
LOC285176	PREDICTED: similar to ribosomal protein L10	XM_937850.1	395.89	176.59	0.45
MGC16121	PREDICTED: hypothetical protein MGC16121	XM_927600.1	260.84	104.40	0.40
SOX9	SRY (sex determining region Y)-box 9	NM_000346.2	717.36	280.22	0.39
WDR79	WD repeat domain 79	NM_018081.1	192.67	74.33	0.38
