# Supplementary material for: SUMOylation of DRIL1 Directs Its Transcriptional Activity Towards Leukocyte Lineage-Specific Genes
Source: PLoS One. 2009 May 14;4(5):e5542. doi: 10.1371/journal.pone.0005542 (PMC2677661; doi:10.1371/journal.pone.0005542)
Supplement: Table S2 — List of Gene Ontology (GO) classes enriched by wt DRIL1. Log2 (odds ratio) of the enrichment of the GOID were obtained using Gene Ontology Enrichment Analysis Software Toolkit ([19], http://omicslab.genetics.ac.cn/GOEAST/). P-value of the significance for the enrichment in the dataset of the listed GOID using multiple-test adjusted false discovery rate (FDR). In bold, GO classes enriched by wt DRIL1 only. (0.05 MB PDF) [file pone.0005542.s002.pdf]

**GO ID**	**term ID**	**log2 (odds ratio)**	**p-value**
GO:0030325	adrenal gland development	5.90	7.85E-04
GO:0035270	endocrine system development	5.90	7.85E-04
GO:0006526	arginine biosynthetic process	5.38	2.17E-03
**GO:0006534**	**cysteine metabolic process**	**5.38**	**2.17E-03**
**GO:0001909**	**leukocyte mediated cytotoxicity**	**5.38**	**1.34E-02**
**GO:0042267**	**natural killer cell mediated cytotoxicity**	**5.38**	**1.34E-02**
**GO:0002228**	**natural killer cell mediated immunity**	**5.38**	**1.34E-02**
GO:0017183	peptidyl-diphthamide biosynthetic process from peptidyl-histidine	5.38	1.34E-02
GO:0017182	peptidyl-diphthamide metabolic process	5.38	1.34E-02
GO:0018202	peptidyl-histidine modification	5.38	1.34E-02
GO:0042448	progesterone metabolic process	5.38	1.34E-02
**GO:0007159**	**leukocyte adhesion**	**5.24**	**2.73E-03**
**GO:0045739**	**positive regulation of DNA repair**	**5.12**	**1.82E-02**
GO:0043603	cellular amide metabolic process	5,00	3.34E-03
**GO:0030593**	**neutrophil chemotaxis**	**5,00**	**3.34E-03**
GO:0000050	urea cycle	5,00	3.34E-03
GO:0019627	urea metabolic process	5,00	3.34E-03
GO:0042632	cholesterol homeostasis	4.90	3.98E-03
GO:0055088	lipid homeostasis	4.90	3.98E-03
GO:0055092	sterol homeostasis	4.90	3.98E-03
GO:0006707	cholesterol catabolic process	4.90	2.31E-02
GO:0030301	cholesterol transport	4.90	2.31E-02
GO:0016127	sterol catabolic process	4.90	2.31E-02
GO:0015918	sterol transport	4.90	2.31E-02
**GO:0006706**	**steroid catabolic process**	**4.80**	**4.72E-03**
**GO:0000097**	**sulfur amino acid biosynthetic process**	**4.80**	**4.72E-03**
GO:0006525	arginine metabolic process	4.70	5.73E-03
GO:0008333	endosome to lysosome transport	4.70	2.77E-02
**GO:0006542**	**glutamine biosynthetic process**	**4.70**	**2.77E-02**
**GO:0060326**	**cell chemotaxis**	**4.53**	**6.84E-03**
**GO:0030595**	**leukocyte chemotaxis**	**4.53**	**6.84E-03**
**GO:0001906**	**cell killing**	**4.53**	**3.15E-02**
GO:0031498	chromatin disassembly	4.53	3.15E-02
**GO:0006337**	**nucleosome disassembly**	**4.53**	**3.15E-02**
**GO:0032986**	**protein-DNA complex disassembly**	**4.53**	**3.15E-02**
**GO:0050900**	**leukocyte migration**	**4.46**	**7.86E-03**
**GO:0015669**	**gas transport**	**4.38**	**8.80E-03**
GO:0048732	gland development	4.38	8.80E-03
**GO:0015671**	**oxygen transport**	**4.38**	**8.80E-03**
**GO:0006835**	**dicarboxylic acid transport**	**4.38**	**3.54E-02**
**GO:0030218**	**erythrocyte differentiation**	**4.38**	**3.54E-02**
**GO:0034101**	**erythrocyte homeostasis**	**4.38**	**3.54E-02**
**GO:0043433**	**negative regulation of transcription factor activity**	**4.38**	**3.54E-02**
GO:0030516	regulation of axon extension	4.38	3.54E-02
GO:0033261	regulation of S phase	4.38	3.54E-02
GO:0007090	regulation of S phase of mitotic cell cycle	4.38	3.54E-02
GO:0006641	triglyceride metabolic process	4.38	3.54E-02
GO:0009084	glutamine family amino acid biosynthetic process	4.29	2.73E-03
GO:0006884	cell volume homeostasis	4.24	4.06E-02
GO:0022600	digestive system process	4.24	4.06E-02
**GO:0048872**	**homeostasis of number of cells**	**4.24**	**4.06E-02**
GO:0048168	regulation of neuronal synaptic plasticity	4.24	4.06E-02
GO:0009070	serine family amino acid biosynthetic process	4.18	1.18E-02
GO:0007212	dopamine receptor signaling pathway	4.12	4.55E-02
**GO:0051100**	**negative regulation of binding**	**4.12**	**4.55E-02**
**GO:0043392**	**negative regulation of DNA binding**	**4.12**	**4.55E-02**
**GO:0051053**	**negative regulation of DNA metabolic process**	**4,00**	**5.04E-02**
**GO:0008156**	**negative regulation of DNA replication**	**4,00**	**5.04E-02**
**GO:0045078**	**positive regulation of interferon-gamma biosynthetic process**	**4,00**	**5.04E-02**
**GO:0008209**	**androgen metabolic process**	**3.90**	**5.63E-02**

GO:0007041	lysosomal transport	3.90	5.63E-02
GO:0007271	synaptic transmission, cholinergic	3.90	5.63E-02
GO:0007611	learning or memory	3.85	1.93E-02
**GO:0050730**	**regulation of peptidyl-tyrosine phosphorylation**	**3.80**	**2.10E-02**
GO:0006700	C21-steroid hormone biosynthetic process	3.80	6.22E-02
GO:0045766	positive regulation of angiogenesis	3.80	6.22E-02
**GO:0006282**	**regulation of DNA repair**	**3.80**	**6.22E-02**
**GO:0009266**	**response to temperature stimulus**	**3.80**	**6.22E-02**
GO:0008652	amino acid biosynthetic process	3.78	4.63E-04
**GO:0000096**	**sulfur amino acid metabolic process**	**3.75**	**2.26E-02**
GO:0006171	cAMP biosynthetic process	3.70	6.77E-02
GO:0006342	chromatin silencing	3.70	6.77E-02
GO:0045814	negative regulation of gene expression, epigenetic	3.70	6.77E-02
**GO:0051054**	**positive regulation of DNA metabolic process**	**3.70**	**6.77E-02**
**GO:0001932**	**regulation of protein amino acid phosphorylation**	**3.66**	**2.48E-02**
GO:0006639	acylglycerol metabolic process	3.62	7.26E-02
**GO:0043450**	**alkene biosynthetic process**	**3.62**	**7.26E-02**
GO:0001502	cartilage condensation	3.62	7.26E-02
**GO:0019370**	**leukotriene biosynthetic process**	**3.62**	**7.26E-02**
**GO:0030101**	**natural killer cell activation**	**3.62**	**7.26E-02**
GO:0006638	neutral lipid metabolic process	3.62	7.26E-02
GO:0051262	protein tetramerization	3.62	7.26E-02
**GO:0045072**	**regulation of interferon-gamma biosynthetic process**	**3.62**	**7.26E-02**
**GO:0032649**	**regulation of interferon-gamma production**	**3.62**	**7.26E-02**
GO:0006662	glycerol ether metabolic process	3.53	7.96E-02
GO:0046486	glycerolipid metabolic process	3.53	7.96E-02
GO:0048705	skeletal system morphogenesis	3.53	7.96E-02
GO:0008207	C21-steroid hormone metabolic process	3.46	8.54E-02
**GO:0006775**	**fat-soluble vitamin metabolic process**	**3.46**	**8.54E-02**
GO:0006379	mRNA cleavage	3.46	8.54E-02
**GO:0007026**	**negative regulation of microtubule depolymerization**	**3.46**	**8.54E-02**
GO:0050770	regulation of axonogenesis	3.46	8.54E-02
**GO:0031114**	**regulation of microtubule depolymerization**	**3.46**	**8.54E-02**
**GO:0006776**	**vitamin A metabolic process**	**3.46**	**8.54E-02**
GO:0042446	hormone biosynthetic process	3.38	3.54E-02
GO:0007589	body fluid secretion	3.38	9.12E-02
GO:0046058	cAMP metabolic process	3.38	9.12E-02
**GO:0031111**	**negative regulation of microtubule polymerization or depolymerization**	**3.38**	**9.12E-02**
**GO:0007422**	**peripheral nervous system development**	**3.38**	**9.12E-02**
**GO:0031110**	**regulation of microtubule polymerization or depolymerization**	**3.38**	**9.12E-02**
**GO:0031399**	**regulation of protein modification process**	**3.31**	**3.73E-02**
GO:0006000	fructose metabolic process	3.31	9.76E-02
GO:0010769	regulation of cell morphogenesis involved in differentiation	3.31	9.76E-02
GO:0000302	response to reactive oxygen species	3.31	9.76E-02
GO:0009064	glutamine family amino acid metabolic process	3.27	1.60E-02
GO:0007050	cell cycle arrest	3.23	8.09E-04
GO:0009309	amine biosynthetic process	3.23	2.73E-03
**GO:0044272**	**sulfur compound biosynthetic process**	**3.21**	**4.31E-02**
**GO:0051090**	**regulation of transcription factor activity**	**3.15**	**4.66E-02**
**GO:0009069**	**serine family amino acid metabolic process**	**3.15**	**4.66E-02**
**GO:0019220**	**regulation of phosphate metabolic process**	**3.12**	**4.81E-02**
**GO:0051174**	**regulation of phosphorus metabolic process**	**3.12**	**4.81E-02**
**GO:0042325**	**regulation of phosphorylation**	**3.12**	**4.81E-02**
GO:0007088	regulation of mitosis	3.09	5.04E-02
GO:0009887	organ morphogenesis	3.09	3.34E-03
GO:0044271	nitrogen compound biosynthetic process	3.03	3.98E-03
**GO:0001525**	**angiogenesis**	**3.02**	**2.48E-02**
GO:0008015	blood circulation	2.95	2.78E-02
GO:0003013	circulatory system process	2.95	2.78E-02
**GO:0051101**	**regulation of DNA binding**	**2.95**	**6.22E-02**
GO:0006869	lipid transport	2.90	1.34E-02
**GO:0008360**	**regulation of cell shape**	**2.90**	**6.64E-02**

GO:0006979	response to oxidative stress	2.88	3.15E-02
**GO:0001568**	**blood vessel development**	**2.86**	**3.15E-02**
**GO:0048514**	**blood vessel morphogenesis**	**2.86**	**3.15E-02**
GO:0034728	nucleosome organization	2.86	1.42E-02
**GO:0007218**	**neuropeptide signaling pathway**	**2.85**	**1.47E-02**
**GO:0001944**	**vasculature development**	**2.83**	**3.41E-02**
GO:0006096	glycolysis	2.8	3.54E-02
GO:0010564	regulation of cell cycle process	2.77	7.76E-02
GO:0043123	positive regulation of I-kappaB kinase/NF-kappaB cascade	2.76	8.28E-03
**GO:0051098**	**regulation of binding**	**2.73**	**8.28E-02**
**GO:0006694**	**steroid biosynthetic process**	**2.69**	**4.15E-02**
GO:0043122	regulation of I-kappaB kinase/NF-kappaB cascade	2.69	9.35E-03
**GO:0051052**	**regulation of DNA metabolic process**	**2.66**	**9.06E-02**
**GO:0007420**	**brain development**	**2.64**	**9.20E-02**
**GO:0008203**	**cholesterol metabolic process**	**2.62**	**4.66E-02**
**GO:0048646**	**anatomical structure formation**	**2.60**	**4.78E-02**
GO:0031497	chromatin assembly	2.60	4.78E-02
GO:0006334	nucleosome assembly	2.60	4.78E-02
GO:0006007	glucose catabolic process	2.59	4.86E-02
GO:0006323	DNA packaging	2.59	2.68E-02
GO:0010740	positive regulation of protein kinase cascade	2.58	1.34E-02
**GO:0016125**	**sterol metabolic process**	**2.43**	**6.48E-02**
GO:0019320	hexose catabolic process	2.42	6.64E-02
**GO:0046365**	**monosaccharide catabolic process**	**2.41**	**6.77E-02**
GO:0006006	glucose metabolic process	2.40	3.59E-02
GO:0009967	positive regulation of signal transduction	2.38	2.24E-02
GO:0010627	regulation of protein kinase cascade	2.38	2.24E-02
**GO:0008202**	**steroid metabolic process**	**2.38**	**1.17E-02**
GO:0046164	alcohol catabolic process	2.37	7.22E-02
GO:0006333	chromatin assembly or disassembly	2.34	4.13E-02
GO:0010647	positive regulation of cell communication	2.33	2.48E-02
GO:0006915	apoptosis	2.32	5.28E-05
GO:0012501	programmed cell death	2.32	5.28E-05
GO:0044275	cellular carbohydrate catabolic process	2.29	8.21E-02
**GO:0008544**	**epidermis development**	**2.26**	**8.58E-02**
**GO:0007417**	**central nervous system development**	**2.23**	**5.08E-02**
GO:0008219	cell death	2.21	1.27E-04
GO:0016265	death	2.21	1.27E-04
GO:0008285	negative regulation of cell proliferation	2.20	1.94E-02
**GO:0007610**	**behavior**	**2.07**	**2.77E-02**
**GO:0008283**	**cell proliferation**	**1.98**	**1.34E-02**
GO:0019318	hexose metabolic process	1.95	9.08E-02
GO:0005996	monosaccharide metabolic process	1.92	9.66E-02
**GO:0007399**	**nervous system development**	**1.89**	**4.29E-02**
**GO:0048513**	**organ development**	**1.83**	**2.73E-03**
GO:0006281	DNA repair	1.82	5.04E-02
GO:0006974	response to DNA damage stimulus	1.80	3.54E-02
GO:0006066	cellular alcohol metabolic process	1.80	3.57E-02
GO:0007267	cell-cell signaling	1.75	4.09E-03
**GO:0006520**	**amino acid metabolic process**	**1.73**	**9.37E-02**
GO:0022402	cell cycle process	1.70	3.54E-02
GO:0034984	cellular response to DNA damage stimulus	1.69	7.25E-02
**GO:0042127**	**regulation of cell proliferation**	**1.67**	**3.69E-02**
GO:0033554	cellular response to stress	1.66	5.39E-02
GO:0051716	cellular response to stimulus	1.65	5.48E-02
GO:0048731	system development	1.63	2.18E-03
**GO:0019752**	**carboxylic acid metabolic process**	**1.57**	**3.59E-02**
**GO:0006082**	**organic acid metabolic process**	**1.56**	**3.71E-02**
GO:0006950	response to stress	1.54	8.60E-04
**GO:0010646**	**regulation of cell communication**	**1.48**	**6.77E-02**
GO:0030154	cell differentiation	1.46	3.15E-02
GO:0007049	cell cycle	1.45	2.48E-02

GO:0009653	anatomical structure morphogenesis	1.45	9.79E-02
GO:0048869	cellular developmental process	1.37	4.29E-02
**GO:0048856**	**anatomical structure development**	**1.36**	**9.35E-03**
**GO:0007275**	**multicellular organismal development**	**1.29**	**4.14E-03**
GO:0050896	response to stimulus	1.29	7.11E-04
GO:0032502	developmental process	1.22	5.14E-04
**GO:0006796**	**phosphate metabolic process**	**1.09**	**8.54E-02**
**GO:0006793**	**phosphorus metabolic process**	**1.09**	**8.54E-02**
**GO:0032501**	**multicellular organismal process**	**1.02**	**6.84E-03**
